# Generalizable, fast, and accurate DeepQSPR with fastprop

**DOI:** 10.1186/s13321-025-01013-4

**Published:** 2025-05-13

**Authors:** Jackson W. Burns, William H. Green

**Affiliations:** Massachusetts Institute of Technology, Cambridge, MA USA

**Keywords:** QSPR, Learned representations, Deep learning, Molecular descriptors

## Abstract

Quantitative Structure–Property Relationship studies (QSPR), often referred to interchangeably as QSAR, seek to establish a mapping between molecular structure and an arbitrary target property. Historically this was done on a target-by-target basis with new descriptors being devised to *specifically* map to a given target. Today software packages exist that calculate thousands of these descriptors, enabling general modeling typically with classical and machine learning methods. Also present today are learned representation methods in which deep learning models generate a target-specific representation during training. The former requires less training data and offers improved speed and interpretability while the latter offers excellent generality, while the intersection of the two remains under-explored. This paper introduces fastprop, a software package and general Deep-QSPR framework that combines a cogent set of molecular descriptors with deep learning to achieve state-of-the-art performance on datasets ranging from tens to tens of thousands of molecules. fastprop provides both a user-friendly Command Line Interface and highly interoperable set of Python modules for the training and deployment of feedforward neural networks for property prediction. This approach yields improvements in speed and interpretability over existing methods while statistically equaling or exceeding their performance across most of the tested benchmarks. fastprop is designed with Research Software Engineering best practices and is free and open source, hosted at github.com/jacksonburns/fastprop.

## Scientific Contribution

fastprop is a QSPR framework that achieves state-of-the-art accuracy on datasets of all sizes without sacrificing speed or interpretability. As a software package fastprop emphasizes Research Software Engineering best practices, reproducibility, and ease of use for experts across domains.

## Introduction

Chemists have long sought a method to relate only the connectivity of a molecule to its corresponding molecular properties. The Quantitative Structure–Property Relationship (QSPR) would effectively solve the forward problem of molecular engineering and enable rapid development. Reviews on the topic are numerous and cover an enormous range of scientific subdomains; a comprehensive review of the literature is beyond the scope of this publication, though the work of Muratov and coauthors [1] provides an excellent starting point for further review. An abridged version of the history behind QSPR is presented here to contextualize the approach of fastprop.

### Historical approaches

Early in the history of computing, limited computational power meant that significant human expertise was required to guide QSPR models toward effectiveness. This materialized in the form of bespoke molecular descriptors - scalar-valued functions which operate on the molecular graph in such a way to reflect relevant structural and electronic information. Examples include rudimentary counting descriptors, the Wiener Index in 1947 [2], Atom-Bond Connectivity indices in 1998 [3], and many others [4]. To this day descriptors are still being developed - the geometric-harmonic-Zagreb degree based descriptors were proposed by Arockiaraj et al. in 2023 [5]. This time consuming technique is of course highly effective but the dispersed nature of this chemical knowledge means that these descriptors are spread out throughout many journals and domains with no single source to compute them all.

The range of regression techniques applied to these descriptors has also been limited. As explained by Muratov et al. [1] QSPR uses linear methods (some of which are now called machine learning) almost exclusively. The over-reliance on this category of approaches may be due to priorities; domain experts seek interpretability in their work, especially given that the inputs are physically meaningful descriptors, and linear methods lend themselves well to this approach. Practice may also have been a limitation, since historically training and deploying neural networks required more computer science expertise than linear methods.

All of this is not to say that Deep Learning (DL) has *never* been applied to QSPR. Applications of DL to QSPR, i.e. DeepQSPR, were attempted throughout this time period but focused on the use of molecular fingerprints rather than descriptors. This may be at least partially attributed to knowledge overlap between deep learning experts and this sub-class of descriptors. Molecular fingerprints are bit vectors which encode the presence or absence of sub-structures in an analogous manner to the “bag of words” featurization strategy common to natural language processing. Experts have bridged this gap to open this subdomain and proved its effectiveness. In Ma and coauthors’ review of DL for QSPR [6], for example, it is claimed that DL with fingerprint descriptors is more effective than with molecular-level descriptors. They also demonstrate that DL outperforms or at least matches classical machine learning methods across a number of ADME-related datasets. The results of the present study demonstrate that molecular-level descriptors actually *are* effective and reaffirm that DL matches or outperforms baselines, in this case linear.

Despite their differences, both classical- and Deep-QSPR shared a lack of generality. Beyond the domains of chemistry where many of the descriptors had been originally devised, models were either unsuccessful or more likely simply never evaluated. As interest began to shift toward the prediction of molecular properties which were themselves descriptors (i.e. derived from quantum mechanics simulations) - to which none of the devised molecular descriptors were designed to be correlated - learned representations (LRs) emerged.

### Shift to learned representations

The exact timing of the transition from fixed descriptors (molecular-level or fingerprints) to LRs is difficult to ascertain [7]. Among the most cited at least is the work of Yang and coworkers in 2019 [8] which laid the groundwork for applying LRs to “Property Prediction” - QSPR by another name. In short, the basic idea is to initialize a molecular graph with only information about its bonds and atoms such as order, aromaticity, atomic number, etc. Then via a Message Passing Neural Network (MPNN) architecture, which is able to aggregate these atom- and bond-level features into a vector in a manner which can be updated, the ‘best’ representation of the molecule is found during training. This method proved highly accurate *and* achieved the generality apparently lacking in descriptor-based modeling. The modern version of the corresponding software package Chemprop (described in [9]) has become a *de facto* standard for property prediction, partially because of the significant development and maintenance effort supporting that open source software project.

Following the initial success of Chemprop numerous representation learning frameworks have been devised, all of which slightly improve performance. The Communicative-MPNN (CMPNN) framework is a modified version of Chemprop with a different message passing scheme to increase the interactions between node and edge features [10]. Uni-Mol incorporates 3D information and relies extensively on transformers [11]. In a “full circle moment” architectures like the Molecular Hypergraph Neural Network (MHNN) have been devised to learn representations for specific subsets of chemistry, in that case optoelectronic properties [12]. Myriad others exist including GSL-MPP (accounts for intra-dataset molecular similarity) [13], SGGRL (trains three representations simultaneously using different input formats) [14], and MOCO (multiple representations and contrastive pretraining) [15].

#### Limitations

Despite the continuous incremental performance improvements, this area of research has serious drawbacks. A thru-theme in these frameworks is the increasing complexity of DL techniques and consequent un-interpretability. This also means that actually *using* these methods to do research on real-world dataset requires varying amounts of DL expertise, creating a rift between domain experts and these methods. Perhaps the most significant failure is the inability to achieve good predictions on small^1^ datasets. This is a long-standing limitation, with the original Chemprop paper stating that linear models are about on par with Chemprop for datasets with fewer than 1000 entries [8].

This limitation is especially challenging because it is a *fundamental* drawback of the LR approach. Without the use of advanced DL techniques like pre-training or transfer learning, the model is essentially starting from near-zero information every time a model is created. This inherently requires larger datasets to allow the model to effectively ‘re-learn’ the chemical intuition which was built in to descriptor- and fixed fingerprint-based representations.

Efforts are of course underway to address this limitation, though no clear universal solution has emerged. One simple but incredibly computationally expensive approach is to use delta learning, which artificially increases dataset size by generating all possible *pairs* of molecules from the available data (thus squaring the size of the dataset). This was attempted by Nalini et al. [16], who used an unmodified version of Chemprop referred to as ‘DeepDelta’ to predict *differences* in molecular properties for *pairs* of molecules. They achieve increased performance over standard LR approaches but *lost* the ability to train on large datasets due to simple runtime limitations. Another promising line of inquiry is the Transformer-CNN model of Karpov et al. [17] which leverages a pre-trained transformer model for prediction, circumventing the need for massive datasets and offering additional benefits in interpretability. This model is unique in that it operates directly on the SMILES representation of the molecule, also offering benefits in structural attribution of predictions. Due to the extensive pre-training this model is often more performant on small datasets than alternatives like ChemProp with the small additional cost of data augmentation. Other increasingly complex approaches are discussed in the outstanding review by van Tilborg et al. [18].

While iterations on LRs and novel approaches to low-data regimes have been in development, the classical QSPR community has continued their work. A turning point in this domain was the release of mordred, a fast and well-developed package capable of calculating more than 1600 molecular descriptors [19]. Critically this package was fully open source and written in Python, allowing it to readily interoperate with the world-class Python DL software ecosystem that greatly benefitted the LR community. Despite previous claims that molecular descriptors *cannot* achieve generalizable QSPR in combination with DL, the opposite is shown here with fastprop.

## Implementation

**Fig. 1:**
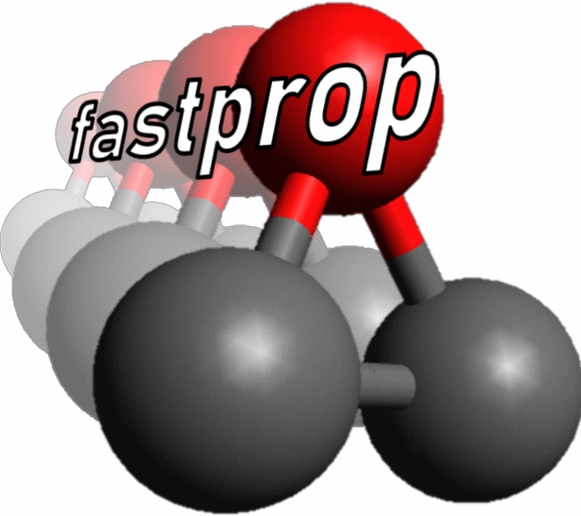
fastprop logo

At its core the fastprop ‘architecture’ is simply the mordred molecular descriptor calculator^2^ [19] connected to a Feedforward Neural Network (FNN) implemented in PyTorch Lightning [20] (Fig. 1) - an existing approach formalized into an easy-to-use, reliable, and correct implementation. fastprop is highly modular for seamless integration into existing workflows and includes an end-to-end Command Line Interface (CLI) for general use. In the latter mode the user simply specifies a set of SMILES [21], a linear textual encoding of molecules, and their corresponding target values. fastprop optionally standardizes input molecule and then automatically calculates and caches the corresponding molecular descriptors with mordred, re-scales both the descriptors and the targets appropriately, and then trains an FNN to predict the indicated targets. By default this FNN is two hidden layers with 1800 neurons each connected by ReLU activation functions, though the configuration can be readily changed via the CLI or configuration file. Multitask regression and multi-label classification are also supported and configurable in the same manner, the former having been shown to significantly improve predictive power in cheminformatics models [22]. fastprop principally owes its success to the cogent set of descriptors assembled by the developers of mordred. Multiple descriptor calculators from the very thorough review by McGibbon et al. [23] could be used instead, though none are as readily interoperable as mordred. Additionally, the ease of training FNNs with modern software like PyTorch Lightning and the careful application of Research Software Engineering best practices make fastprop as user friendly as the best-maintained alternatives.

Fitting to molecular descriptors requires careful attention given that they can be highly correlated, often have enormous outliers exacerbated with re-scaling, and can be missing or infinite for some species. fastprop includes extensive, configurable data pre-processing steps to accommodate these limitations. First and foremost, users can opt to use a subset of 947 less-correlated ($r<0.95$ on QM8 [24]) mordred descriptors, though this is usually unnessecary. Before training, all features are standardized to have mean of zero and variance of one. Missing features are then set to zero, equivalent to imputing with the mean value. Finally, descriptors having values larger than ± 3 are set to ± 3, analogous to Winsorization based on 3 standard deviations from the mean. Other common pre-processing transformations, such as the $log_{10}$ function, are easily implemented when using fastprop as a Python module.

This trivially simple idea has been alluded to in previous published work but neither described in detail nor lauded for its generalizability or accuracy. Comesana and coauthors, based on a review of the biofuels property prediction landscape, claimed that methods (DL or otherwise) using large numbers of molecular descriptors were unsuccessful, instead proposing a feature selection method [25]. As a baseline in a study of photovoltaic property prediction, Wu et al. reported using the mordred descriptors in combination with both a Random Forest and an Artificial Neural Network, though in their hands the performance is worse than their bespoke model and no code is available for inspection [26].

Others have also incorporated mordred descriptors into their modeling efforts, though none with a simple FNN as described above. Esaki and coauthors started a QSPR study with mordred descriptors for a dataset of small molecules, but via an enormously complex modeling pipeline (using only linear methods) removed all but 53 [27]. Yalamanchi and coauthors used DL on mordred descriptors as part of a two-headed representation, but their network architecture was sequential hidden layers *decreasing* in size to only 12 features [28] as opposed to the constant 1800 in fastprop.

The reason fastprop stands out from these studies and contradicts previous reports is for the simple reason that it works. As discussed at length in the Results & Discussion section, this approach statistically matches or exceeds the performance of leading LR approaches on common benchmark datasets and bespoke QSPR models on small real-world datasets. fastprop also overcomes the limitations of LRs discussed above. The FNN architecture and use of physically meaningful molecular descriptors enables the application of SHAP [29], a common tool for feature importance analysis (see Interpretability). The simplicity of the framework enables domain experts to apply it easily and makes model training dramatically faster than LRs. Most importantly this approach is successful on the *smallest* of real-world datasets. By starting from such an informed initialization the FNN can be readily trained on datasets with as few as *forty* training examples (see PAHs).

### Example usage

fastprop is built with ease of use at the forefront of design. To that end, input data is accepted in the immensely popular Comma-Separated Values (CSV) format, editable with all modern spreadsheet editors and completely platform independent. An example specify some properties for benzene is shown below, including its SMILES string:
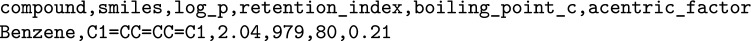


fastprop itself is accessed via the command line interface, with configuration parameters passed as either command line arguments or in an easily editable configuration file:
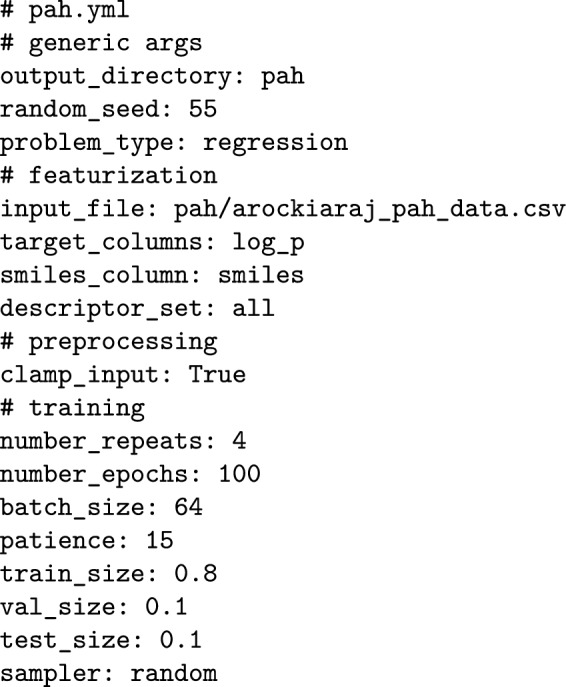


Training, prediction, and feature importance are then readily accessible via the commands fastprop train, fastprop predict, or fastprop shap, respectively. The fastprop GitHub repository contains a Jupyter notebook runnable from the browser via Google colab which allows users to actually execute the above example, which is also discussed at length in the PAHs section, as well as further details about each configurable option.

## Results and discussion

There are a number of established molecular property prediction benchmarks commonly used in LR studies, especially those standardized by MoleculeNet [30]. Principal among them are QM8 [24] and QM9 [31], often regarded as *the* standard benchmark for property prediction. These are important benchmarks and QM9 is included for completeness, though the enormous size and rich coverage of chemical space in the QM9 dataset means that nearly all model architectures are highly accurate, including fastprop.

Real world experimental datasets, particularly those common in QSPR studies, often number in the hundreds. To demonstrate the applicability of fastprop to these regimes, many smaller datasets are selected including some from the QSPR literature that are not established benchmarks. These studies relied on more complex and slow modeling techniques (ARA) or the design of a bespoke descriptor (PAHs) and have not yet come to rely on learned representations as a go-to tool. In these data-limited regimes where LRs sometimes struggle, fastprop and its intuition-loaded initialization are highly powerful. To emphasize this point further, the benchmarks are presented in order of dataset size, descending.

Two additional benchmarks showing the limitations of fastprop are included after the main group of benchmarks: Fubrain and QuantumScents. The former demonstrates how fastprop can outperform LRs but still trail approaches like delta learning. The later is a negative result showing how fastprop can fail on especially difficult, atypical targets.

All of these fastprop benchmarks are reproducible, and complete instructions for installation, data retrieval and preparation, and training are publicly available on the fastprop GitHub repository at github.com/jacksonburns/fastprop.

### Benchmark methods

The method for splitting data into training, validation, and testing sets varies on a per-study basis and is described in each sub-section. Sampling is performed using the astartes package [32] which implements a variety of sampling algorithms and is highly reproducible. For datasets containing missing target values or invalid SMILES strings, those entries were dropped, as is the default behavior of fastprop.

Results for fastprop are reported as the average value of a metric and its standard deviation across a number of repetitions (repeated re-sampling of the dataset). The number of repetitions is chosen to either match referenced literature studies or else increased from two until the performance no longer meaningfully changes. Note that this is *not* the same as cross-validation. Each section also includes the performance of a zero-layer (i.e. linear regression) network as a baseline to demonstrate the importance of non-linearity in a deep NN.

For performance metrics retrieved from literature it is assumed that the authors optimized their respective models to achieve the best possible results; therefore, fastprop metrics are reported after model optimization using the fastprop train... --optimize option. When results are generated for this study using Chemprop, the default settings are used except that the number of epochs is increased to allow the model to converge and batch size is increased to match dataset size and speed up training. Chemprop was chosen as a point of comparison throughout this study since it is among the most accessible and well-maintained software packages for molecular machine learning.

When reported, execution time is as given by the unix time command using Chemprop version 1.6.1 on Python 3.8 and includes the complete invocation of Chemprop, i.e. time chemprop_train..... The insignificant time spent manually collating Chemprop results (Chemprop does not natively support repetitions) is excluded. fastprop is run on version 1.0.6 using Python 3.11 and timing values are reported according to its internal time measurement which was verified to be nearly identical to the Unix time command. The coarse comparison of the two packages is intended to emphasize the scaling of LRs and Deep-QSPR and that fastprop is, generally speaking, much faster. All models trained for this study were run on a Dell Precision series laptop with an NVIDIA Quadro RTX 4000 GPU and Intel Xeon E-2286 M CPU.

Because the diversity of methods across these different datasets complicates inter-dataset comparisons, an additional set of benchmarks using an identical method across all datasets is included in Additional File 1, Table S1. This benchmark compares fastprop, Chemprop, and the aforementioned Transformer-CNN, which is especially suitable for the small datasets included therein. No long-form commentary on benchmark results is provided, though the conclusions are largely the same as those shown here.

#### Performance metrics

The evaluation metrics used in each of these benchmarks are chosen to match literature precedent, particularly as established by MoleculeNet [30], where available. It is common to use scale-dependent metrics that require readers to understand the relative magnitude of the target variables. The authors prefer more readily interpretable metrics such as (Weighted) Mean Absolute Percentage Error (W/MAPE) and are included where relevant.

All metrics are defined according to their typical formulae which are readily available online and are implemented in common software packages. Those presented here are summarized below, first for regression:

- Mean Absolute Error (MAE): Absolute difference between predictions and ground truth averaged across dataset; scale-dependent.
- Root Mean Squared Error (RMSE): Absolute differences *squared* and then averaged; scale-dependent.
- Mean Absolute Percentage Error (MAPE): MAE except that differences are relative (i.e. divided by the ground truth); scale-independent, range 0 (best) and up.
- Weighted Mean Absolute Percentage Error (WMAPE): MAPE except the average is a weighted average, where the weight is the magnitude of the ground truth; scale-independent, range 0 (best) and up.
- Coefficient of Determination (R2): Proportion of variance explained; scale-independent, range 0 (worst) to 1 (best).

and classification:

- Area Under the Receiver Operating Curve (AUROC, AUC, or ROC-AUC): Summary statistic combining all possible classification errors; scale-independent, range 0.5 (worst, random guessing) to 1.0 (perfect classifier).
- Accuracy: Fraction of correct classifications, expressed as a percentage; scale-independent, range 0 (worst) to 100 (perfect classifier).

### Benchmark results

See Table 1 for a summary of all the results. Subsequent sections explore each in greater detail. For benchmarks with statistics, practically significant best performers are shown in bold.

**Table 1:** Summary of benchmark results, best state-of-the-art method vs. fastprop and Chemprop

Benchmark	Samples	Metric	SOTA	fastprop	Chemprop	p
QM9	133,885	MAE	0.0047$^a$	0.0060	0.0081$^a$	~
Pgp	1,275	AUROC	0.94$^b$	0.90	0.89$^b$	~
ARA	842	Accuracy	91$^c$	88	82*	0.083
Flash	632	RMSE	13.2$^d$	**13.0**	21.2*	0.021
YSI	442	MAE	22.3$^e$	25.0	28.9*	0.29
PAH	55	R2	0.96$^f$	**0.97**	0.59*	0.0012

a [11] b [33] c [34] d [35] e [36] f [5] * These reference results were generated for this study

Statistical comparisons of fastprop to Chemprop (shown in the p column) are performed using the non-parametric Wilcoxon-Mann-Whitney Test as implemented in GNumeric. Values are only shown for results generated in this study which are known to be performed using the same methods. Only the results for Flash and PAH are statistically significant at 95% confidence (p<0.05), see benchmark-specific subsections for confidence intervals.

#### QM9

Originally described in Scientific Data [31] and perhaps the most established property prediction benchmark, Quantum Machine 9 (QM9) provides quantum mechanics derived descriptors for many small molecules containing one to nine heavy atoms, totaling 133,885. The data was retrieved from MoleculeNet [30] in a readily usable format. As a point of comparison, performance metrics are retrieved from the paper presenting the UniMol architecture [11] previously mentioned. In that study they trained on only three especially difficult targets (homo, lumo, and gap) using scaffold-based splitting (a more challenging alternative to random splitting), reporting mean and standard deviation across 3 repetitions.

fastprop achieves 0.0060 ± 0.0002 mean absolute error, whereas Chemprop achieves 0.00814 ± 0.00001 and the UniMol framework manages 0.00467 ± 0.00004. This places the fastprop framework ahead of previous learned representation approaches but still trailing UniMol. This is not completely unexpected since UniMol encodes 3D information from the dataset whereas Chemprop and fastprop use only 2D. Future work could evaluate the use of 3D-based descriptors to improve fastprop performance in the same manner that UniMol has with LRs. All methods are better than a purely linear model trained on the molecular descriptors, which manages only a 0.0095 ± 0.0006 MAE.

#### Pgp

First reported in 2011 by Broccatelli and coworkers [37], this dataset has since become a standard benchmark and is included in the Therapeutic Data Commons (TDC) [38] model benchmarking suite. The dataset maps 1,275 small molecule drugs to a binary label indicating if they inhibit P-glycoprotein (Pgp). TDC serves this data through a Python package, but due to installation issues the data was retrieved from the original study instead. The recommended splitting approach is a 70/10/20 scaffold-based split which is done here with 4 replicates.

The model in the original study uses a molecular interaction field but has since been surpassed by other models. According to TDC the current leader [33] on this benchmark has achieved an AUROC of 0.938 ± 0.002^3^. On the same leaderboard Chemprop [8] achieves 0.886 ± 0.016 with the inclusion of additional molecular features. fastprop yet again approaches the performance of the leading methods and outperforms Chemprop, here with an AUROC of 0.903 ± 0.033 and an accuracy of 83.6 ± 4.6%. Remarkably, the linear QSPR model outperforms both Chemprop and fastprop, approaching the performance of the current leader with an AUROC of 0.917 ± 0.016 and an accuracy of 83.8 ± 3.9%.

#### ARA

Compiled by Schaduangrat et al. in 2023 [34], this dataset maps 842 small molecules to a binary label indicating if the molecule is an Androgen Receptor Antagonist (ARA). The reference study introduced DeepAR, a highly complex modeling approach, which achieved an accuracy of 91.1% and an AUROC of 0.945.

For this study an 80/10/10 random splitting is repeated four times on the dataset since no analogous split to the reference study can be determined. Chemprop takes 16 min and 55 s to run on this dataset and achieves only 82.4 ± 2.0% accuracy and 0.898 ± 0.022 AUROC. fastprop takes only 1 min and 54 s (1 min and 39 s for descriptor calculation) and is competitive with the reference study in performance, achieving a 88.2 ± 3.7% accuracy and 0.935 ± 0.034 AUROC. The purely linear QSPR model falls far behind these methods with a 71.8 ± 6.6% accuracy and 0.824 ± 0.052 AUROC.

#### Flash

First assembled and fitted to by Saldana and coauthors [35] the dataset (Flash) includes around 632 entries, primarily alkanes and some oxygen-containing compounds, and their literature-reported flash point. The reference study reports the performance on only one repetition, but manually confirms that the distribution of points in the three splits follows the parent dataset. The split itself was a 70/20/10 random split, which is repeated four times for this study.

Using a complex multi-model ensembling method, the reference study achieved an RMSE of 13.2, an MAE of 8.4, and an MAPE of 2.5%. fastprop matches this performance, achieving 13.0 ± 2.0 RMSE, 9.0 ± 0.5 MAE, and 2.7% ± 0.1% MAPE. Chemprop, however, struggles to match the accuracy of either method - it manages an RMSE of 21.2 ± 2.2, an MAE of 13.8 ± 2.1, and a MAPE of 3.99 ± 0.36%. This is worse than the performance of the linear QSPR model, with an RMSE of 16.1 ± 4.0, an MAE of 11.3 ± 2.9, and an MAPE of 3.36 ± 0.77%.

fastprop dramatically outperforms both methods in terms of training time. The reference model required significant manual intervention to create a model ensemble, so no single training time can be fairly identified. fastprop arrived at the indicated performance without any manual intervention in only 30 s, 13 of which were spent calculating descriptors. Chemprop, in addition to not reaching the same level of accuracy, took 5 min and 44 s to do so - more than ten times the execution time of fastprop.

#### YSI

Assembled by Das and coauthors [36] from a collection of other smaller datasets, this dataset maps 442 molecular structures to a unified-scale Yield Sooting Index (YSI), a molecular property of interest to the combustion community. The reference study performs leave-one-out cross validation to fit a per-fragment contribution model, effectively a training size of >99%, without a holdout set. Though this is not standard practice and can lead to overly optimistic reported performance, the results will be carried forward regardless. The original study did not report overall performance metrics, so they have been re-calculated for this study using the predictions made by the reference model as provided on GitHub^4^. For comparison fastprop and Chemprop use a more typical 60/20/20 random split and 8 repetitions. Results are summarized in Table 2.

**Table 2:** Accuracy of YSI predictions from Reference model [36], Linear QSPR model, fastprop, and Chemprop

Model	MAE	RMSE	WMAPE
Reference	22.3	50	14
fastprop	25.0 ± 5.2	52 ± 20	13.6 ±1.3
Chemprop	28.9 ± 6.5	63 ± 14	16.4 ± 3.0
Linear	82 ± 39	180 ± 120	47.0 ± 2.3

fastprop again outperforms Chemprop, in this case approaching the overly-optimistic performance of the reference model. Taking into account that reference model has been trained on a significantly larger amount of data, this performance is admirable. Also notable is the difference in training times. Chemprop takes 7 min and 2 s while fastprop completes in only 42 s, again a factor of ten faster. The linear QSPR model fails entirely, performing dramatically worse than all other models.

#### PAH

Originally compiled by Arockiaraj et al. [5] the Polycyclic Aromatic Hydrocarbons (PAH) dataset contains water/octanol partition coefficients (logP) for 55 polycyclic aromatic hydrocarbons ranging in size from napthalene to circumcoronene. This size of this benchmark is an ideal case study for the application of fastprop. Using expert insight the reference study designed a novel set of molecular descriptors that show a strong correlation to logP, with correlation coefficients ranging from 0.96 to 0.99 among the various new descriptors.

For comparison, fastprop and Chemprop are trained using 8 repetitions of a typical 80/10/10 random split - only **44** molecules in the training data. fastprop matches the performance of the bespoke descriptors with a correlation coefficient of 0.972 ± 0.025. This corresponds to an MAE of 0.19 ± 0.10 and an MAPE of 2.5 ± 1.5%. Chemprop effectively fails on this dataset, achieving a correlation coefficient of only 0.59 ± 0.24, an MAE of 1.04 ± 0.33 (one anti-correlated outlier replicate removed). This is worse even than the purely linear QSPR model, which manages a correlation coefficient of 0.78 ± 0.22, an MAE of 0.59 ± 0.22, and an RMSE of 0.75 ± 0.32. Despite the large parameter size of the fastprop model relative to the training data, it readily outperforms Chemprop in the small-data limit.

For this unique dataset, execution time trends are inverted. fastprop takes 1 min and 43 s, of which 1 min and 31 s were spent calculating descriptors for these unusually large molecules. Chemprop completes in 1 min and 16 s, faster overall but much slower compared with the training time of fastprop without descriptor calculation.

## Limitations and future work

### Negative results

The fastprop framework is not without its drawbacks. The two subsequent sections explore in greater detail two specific cases where fastprop loses out to existing methods, but some general notes about out-of-distribution predictions and overfitting are also included here. Like all machine learning methods, fastprop is not intended to make predictions outside of its training feature space. The use of molecular descriptors, which can become out-of-distribution, may exacerbate this problem but fastprop can optionally winsorize the descriptors to counteract this issue. Additionally, hyperparameter optimization of machine learning models in cheminformatics has been known to cause overfitting [39], especially on small datasets. Users should be cautious when optimizing fastprop models and rely on defaults when possible.

#### Delta learning with fubrain

First described by Esaki and coauthors, the Fraction of Unbound Drug in the Brain (Fubrain) dataset is a collection of 254 small molecule drugs and their corresponding experimentally measured unbound fraction in the brain, a critical metric for drug development [27]. This specific target in combination with the small dataset size makes this benchmark highly relevant for typical QSPR studies, particular via delta learning. DeepDelta [16] performed a 90/0/10 cross-validation study of the Fubrain dataset in which the training and testing molecules were intra-combined to generate all possible pairs and then the differences in the property^5^ were predicted, rather than the absolute values, increasing the amount of training data by a factor of 300.

DeepDelta reported an RMSE of 0.830 ± 0.023 at predicting differences, whereas a typical Chemprop model trained to directly predict property values was only able to reach an RMSE of 0.965 ± 0.09 when evaluated on its capacity to predict property differences. fastprop is able to outperform Chemprop, though not DeepDelta, achieving an RMSE of 0.930 ± 0.029 when using the same splitting procedure above. It is evident that delta learning is still a powerful technique for regressing small datasets.

For completeness, the performance of Chemprop and fastprop when directly predicting the unbound fraction are also compared to the original study by Esaki and coauthors. Using both cross validation and and external test sets, they had an effective training/validation/testing split of 0.64/0.07/0.28 which will be repeated 4 times here for comparison. They used mordred descriptors in their model but as is convention they strictly applied linear modeling methods. All told, their model achieved an RMSE of 0.53 averaged across all testing data. In only 39 s, of which 31 are spent calculating descriptors, fastprop far exceeds the reference model with an RMSE of 0.207 ± 0.024. This also surpasses Chemprop, itself outperforming the reference model with an RMSE of 0.223 ± 0.036.

#### fastprop fails on QuantumScents

Compiled by Burns and Rogers [40], this dataset contains approximately 3.5k SMILES and 3D structures for a collection of molecules labeled with their scents. Each molecule can have any number of reported scents from a possible 113 different labels, making this benchmark a a Quantitative Structure-Odor Relationship. Due to the highly sparse nature of the scent labels a unique sampling algorithm (Szymanski sampling [41]) was used in the reference study and the exact splits are replicated here for a fair comparison.

In the reference study, Chemprop achieved an AUROC of 0.85 with modest hyperparameter optimization and an improved AUROC of 0.88 by incorporating the atomic descriptors calculated as part of QuantumScents. fastprop is incapable of incorporating atomic features, so they are not included. Using only the 2D structural information, fastprop falls far behind the reference study with an AUROC of only 0.651 ± 0.005. Even when using the high-quality 3D structures and calculating additional descriptors (demonstrated in the GitHub repository), the performance does not improve.

The exact reason for this failure is unknown. Possible reasons include that the descriptors in mordred are simply not correlated with this target, and thus the model struggles to make predictions. This is a fundamental drawback of this fixed representation method - whereas a LR could adapt to this unique target, fastprop fails.

### Execution time

fastprop is consistently faster to train than Chemprop when using a GPU, helping exploit the ‘time value’ of data. Note that due to the large size of the FNN in fastprop it can be slower than small Chemprop models when training on a CPU since Chemprop uses a much smaller FNN and associated components.

There is a clear performance improvement to be had by reducing the number of descriptors to a subset of only the most important. Future work can address this possibility to decrease time requirements for both training by reducing network size and inference by decreasing the number of descriptors to be calculated for new molecules. This has *not* been done in this study for two reasons: (1) to emphasize the capacity of the DL framework to effectively perform feature selection on its own via the training process, de-emphasizing unimportant descriptors; (2) as discussed above, training time is small compared to dataset generation time, or even compared to to the time it takes to compute the descriptors using mordred.

### Coverage of descriptors

fastprop is fundamentally limited by the types of chemicals which can be uniquely described by the mordred package. Domain-specific additions which are not just derived from the descriptors already implemented will be required to expand its application to new domains. To facilitate this use case fastprop allows users to pass pre-computed descriptors from the CLI. This allows seamless interoperation with other user-developed descriptors or other molecular descriptor calculators.

For example, in its current state mordred does not include any connectivity based-descriptors that reflect the presence or absence of stereocenters. While some of the 3D descriptors it implements could implicitly reflect sterochemistry, more explicit descriptors like the Stereo Signature Molecular Descriptor [42] may prove helpful in the future if re-implemented in mordred.

### Interpretability

Though not discussed here for the sake of length, fastprop contains the functionality to perform feature importance studies on trained models. By using SHAP values [29] once can assign a scalar ‘importance’ to each of the input features with respect to the target value, such as molecular weight having a significant positive impact on boiling point in alkanes. Experts users can leverage this information to guide molecular design and optimization or inform future lines of inquiry. Via the fastprop CLI users can train a model and then use fastprop shap to analyze the resulting trained network. fastprop shap will then generate diagrams to visualize the SHAP values.

### Availability

- Project name: fastprop
- Project home page: github.com/jacksonburns/fastprop
- Operating system(s): Platform independent
- Programming language: Python
- Other requirements: pyyaml, lightning, mordredcommunity, astartes
- License: MIT

## Supplementary Information

Supplementary material 1.

## Data Availability

fastprop is Free and Open Source Software; anyone may view, modify, and execute it according to the terms of the MIT license. See github.com/jacksonburns/fastprop for more information. All data used in the Benchmarks shown above is publicly available under a permissive license. See the benchmarks directory at the fastprop GitHub page for instructions on retrieving each dataset and preparing it for use with fastprop, where applicable.

## Footnotes

1 What constitutes a ‘small’ dataset is decidedly *not* agreed upon. For the purposes of this study, it will be used to refer to datasets with ~1000 molecules or fewer, which the authors believe better reflects the size of real-world datasets.

2 The original mordred package is no longer maintained. fastprop uses a fork of mordred called mordredcommunity that is maintained by community-contributed patches (see github.com/JacksonBurns/mordred-community). Others have re-implemented the mordred calculator as osmordred which can be used in fastprop via the CLI.

3 See the TDC Pgp leaderboard https://tdcommons.ai/benchmark/admet_group/03pgp/.

4 Predictions are available at this permalink to the CSV file on GitHub.

5 Although the original Fubrain study reported untransformed fractions, the DeepDelta authors confirmed via GitHub that DeepDelta was trained on log base-10 transformed fraction values, which is replicated here.
