# Supplementary material for: Generalizable, fast, and accurate DeepQSPR with fastprop
Source: J Cheminform. 2025 May 13;17:73. doi: 10.1186/s13321-025-01013-4 (PMC12076996; doi:10.1186/s13321-025-01013-4)
Supplement: Supplementary file 1 — Supplementary material 1. [file 13321_2025_1013_MOESM1_ESM.pdf]

Additional File 1

Table S1

The following table shows the performance (see Metric column) for Chemprop, Transformer-CNN, and `fastprop` across various open datasets.

For further information about exact software versions for reproducing these results, see this GitHub repository which hosts both the training code and the results: github.com/JacksonBurns/fastprop-benchmark. All datasets are retrieved from MoleculeNet, except Tox24 which was retrieved from OChem.eu - direct links are provided in the aforementioned repository. The version of Transformer-CNN used is that referenced in the original publication, as made available on GitHub at github.com/bigchem/transformer-cnn. A more user-friendly version with updated dependencies has been made available on GitHub at github.com/osmoai/transformer-CNN and may be preferable for users looking to use Transformer-CNN on their own data.

Benchmarks are sorted by size, descending. Each result is the average and standard deviation across five randomly selected train/val/test splits of 0.70/0.10/0.20. All models were trained with their default settings, i.e. no hyperparameter optimization was performed. Augmentation at training or inference time was not performed. Transformer-CNN suggests using augmentation to correct for the impact of multiple valid SMILES encodings per a given molecule, but because the impact of augmentation on ChemProp and `fastprop` is not understood it is neglected. For Mean Absolute Error (MAE) and Root Mean Squared Error (RMSE) lower is better and for Receiver Operating Characteristic Area Under the Curve (ROC-AUC) higher is better. The Tukey test is used to check for pairwise statistical differences between the three models. The best performing model(s) which are statistically significantly different from the others are shown in bold.

Dataset	Entries	Metric	Chemprop	Transformer-CNN	fastprop
HIV	41,127	ROC-AUC	**0.828(0.015)**	0.56(0.13)	**0.784(0.020)**
QM8	21,786	MAE	**0.0056(0.0001)**	0.0136(0.0004)	0.0164(0.0002)
QM7	6,834	MAE	68.1(2.7)	62.4(2.2)	**57.1(2.8)**
Lipophilicity	4,200	RMSE	**0.597(0.033)**	0.702(0.030)	0.736(0.020)
BBBP	2,050	ROC-AUC	0.918(0.016)	**0.9650(0.0032)**	0.903(0.013)
BACE	1,513	ROC-AUC	0.856(0.010)	**0.899(0.016)**	**0.878(0.015)**
ClinTox	1,484	ROC-AUC	**0.877(0.035)**	**0.9814(0.0083)**	0.64(0.13)
SIDER	1,427	ROC-AUC	**0.662(0.039)**	**0.612(0.031)**	**0.629(0.016)**
Tox24	1,212	RMSE	**26.3(1.9)**	**25.0(1.1)**	**27.4(1.7)**
ESOL	1,128	RMSE	**0.683(0.044)**	**0.701(0.057)**	**0.81(0.15)**
FreeSolv	642	RMSE	**1.32(0.23)**	**1.50(0.23)**	**1.29(0.15)**
